# Fibrous Dysplasia of Proximal Femur: A Case Report of Treatment With Single-Stage Valgus Osteotomy With Dynamic Hip Screw and Fibular Strut Graft

**DOI:** 10.7759/cureus.21496

**Published:** 2022-01-22

**Authors:** Avin Vyas, Anil Godara, Naveen Kumar, Shrey Singhal, Dhritobroto Bhattacherjee

**Affiliations:** 1 Orthopedics, Maharishi Markandeshwar University, Mullana, Ambala, IND; 2 Orthopedics, Maharishi Markandeshwar Institute of Medical Sciences and Research, Maharishi Markandeshwar University, Mullana, Ambala, IND; 3 Orthopedics and Traumatology, Maharishi Markandeshwar Institute of Medical Sciences and Research, Ambala, IND

**Keywords:** single-stage osteotomy, shepherd’s crook deformity, fibular strut graft, fibrous dysplasia, benign bone lesion

## Abstract

Fibrous dysplasia is a rare benign intramedullary fibro-osseous lesion characterized by fibro-osseous proliferation with intervening areas of normal or immature bone. It can either be a monostotic or a polyostotic presentation. The etiology of fibrous dysplasia has been linked with a missense mutation in the GNAS1 gene on chromosome 20. The most common fibrous dysplasia is first diagnosed in children or young adults. There is no gender predilection. Overall, fibrous dysplasia constitutes 5% of all benign bone lesions.The monostotic form is the most frequent, accounting for 75% to 80% of fibrous dysplasia cases.

We report a case of unilateral monostotic fibrous dysplasia in a 30-year-old male in the proximal femur with Shepherd's crook deformity. The patient underwent a single-stage procedure of curettage of lesion and valgus osteotomy with dynamic hip screw (DHS) fixation and fibular strut graft. The lesion resulted in alteration of hip joint anatomy with a decrease in the neck-shaft angle to 114 degrees and leading to coxa vara. After surgical correction, the neck-shaft angle was restored to 130 degrees. The patient was followed up in the outpatient department (OPD), x-rays were taken, and signs of radiological healing were seen at three months. Partial weight-bearing was allowed at three months postoperatively and full weight-bearing at six months with no restriction in the activity. After six months, the patient was able to perform all activities without any difficulty, and shortening of 1.5 cm was compensated with footwear modification. No evidence of recurrence was noted in the follow-up x-ray.

Fibrous dysplasia of proximal femur treated with curettage and bone grafting and supported with an osteotomy to correct mechanical alignment provides excellent results. DHS, though old hardware, provides a versatile option to support osteotomy and helps in maintaining the correction. To support the neck femur after curettage, the fibula strut graft provides an excellent option. When the procedure is done in a single stage, it gives good functional and radiological outcomes along with early rehabilitation.

## Introduction

Fibrous dysplasia (FD) is a rare benign intramedullary fibro-osseous lesion characterized by the presence of immature fibrous tissue in place of the normal architecture of bone originally described by Lichtenstein [1] in 1938 and later confirmed by Lichtenstein and Jaffe in 1942 [2]. The two forms of FD are the monostotic or polyostotic types with similar histological characteristics. The etiology of FD has been linked with a mutation in the GNAS1 gene on chromosome 20 [3]. The most common FD is first diagnosed in children or young adults.

McCune-Albright syndrome, a rare entity with estimated prevalence ranges between 1/100,000 and 1/1,000,000, is defined by polyostotic FD, café-au-lait spots, and precarious puberty endocrine dysfunction like hyperthyroidism, excess growth hormones, and renal phosphate wasting [4]. The monostotic form is 7.6 times more frequent, but both variants show no predilection for gender [5]. Pain is the most common clinical feature of the lesion if not associated with pathological fracture. The severity of pain was higher in patients with lesions of the lower extremities and ribs compared with upper extremity or craniofacial lesions.

Other forms of FD (polyostotic/McCune-Albright syndrome) were more often associated with pain, often severe [6]. The clinical spectrum of the disease ranges from simple bone enlargement or bone pain to pathological fractures and deformities of bone. Monostotic FD cases are often asymptomatic, which can be followed periodically with assessment for new symptoms and radiographs or sometimes may be detected as an incidental finding. Treatment is not required in asymptomatic cases. The mainstay of treatment for FD is surgical, but antiresorptive medication (bisphosphonates, anti-receptor activator of nuclear factor-kappaB ligand [anti-RANKL] antibody) aims at decreasing the local increase in bone turnover in the management of FD, thereby potentially decreasing or preventing the expansion of lesions, controlling the symptoms, and decreasing the risk for deformities and fractures if detected at an early stage [7].

Surgical management is as outlined by Enneking et al., which involved curettage of lesion and using bone graft to fill the defect for prevention of fracture and deformity [8]. A detailed guide to treating FD with Shepherd's crook deformity with fracture of neck femur was published by Dheenadhayalan et al. [9]. Recurrence of lesions can occur if the lesion is managed only with curettage and bone grafting. We report a case of unilateral monostotic FD in a 30-year-old male with Shepherd's crook deformity along with pathological neck femur fracture managed with single-stage valgus osteotomy with combined dynamic hip screw (DHS) and fibular strut graft.

## Case presentation

**Figure 1:**
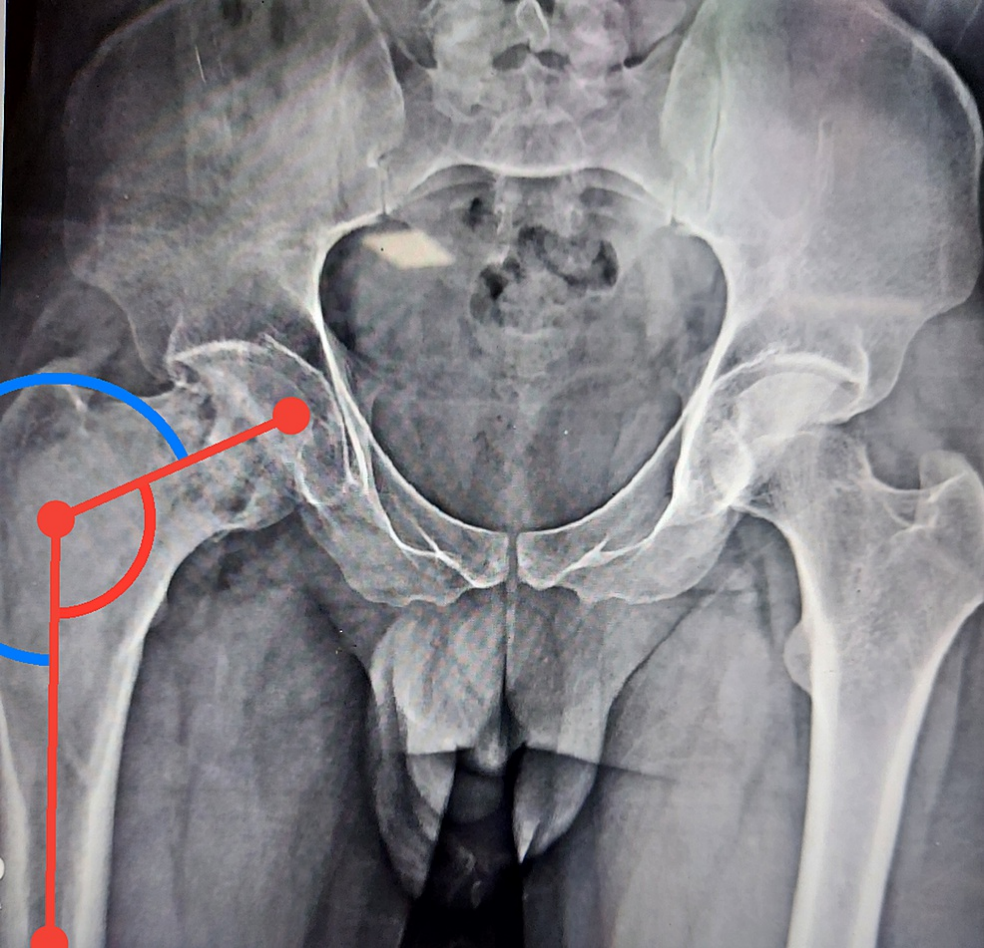
Preoperative x-ray showing Shepherd's crook deformity with neck-shaft angle of 114 degrees

**Figure 2:**
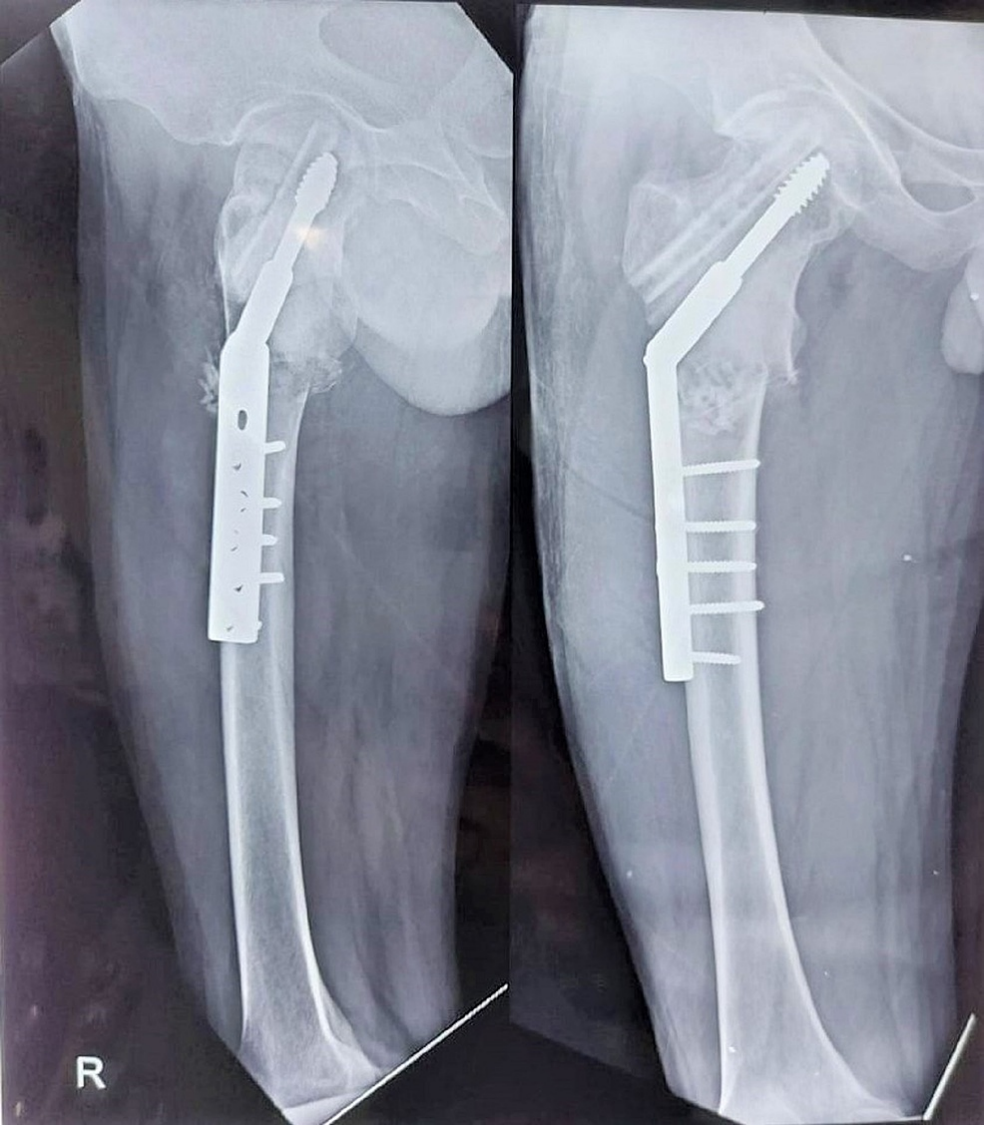
Immediate postoperative x-ray

A 30-year-old male patient presented to us with difficulty in walking, a limp in the right lower limb, and difficulty in squatting for the past six months. The patient suffered a fall from a ladder and sustained an injury to the right hip six months back, for which, initially, the patient took treatment from a traditional medicine practitioner. The patient presented to us six months later with the above-mentioned symptoms. After a thorough clinico-radiological examination of the patient, the diagnosis of FD with Shepherd's crook deformity hip was established. There was no evidence of any endocrine disturbance on metabolic bone profile evaluation or altered pigmentation. The patient underwent a single-stage procedure of valgus osteotomy with DHS fixation, and a fibular strut graft was planned. The preoperative neck-shaft angle calculated was 114 degrees, and after correction, the neck-shaft angle was 130 degrees (Figures 1, 2).

**Figure 3:**
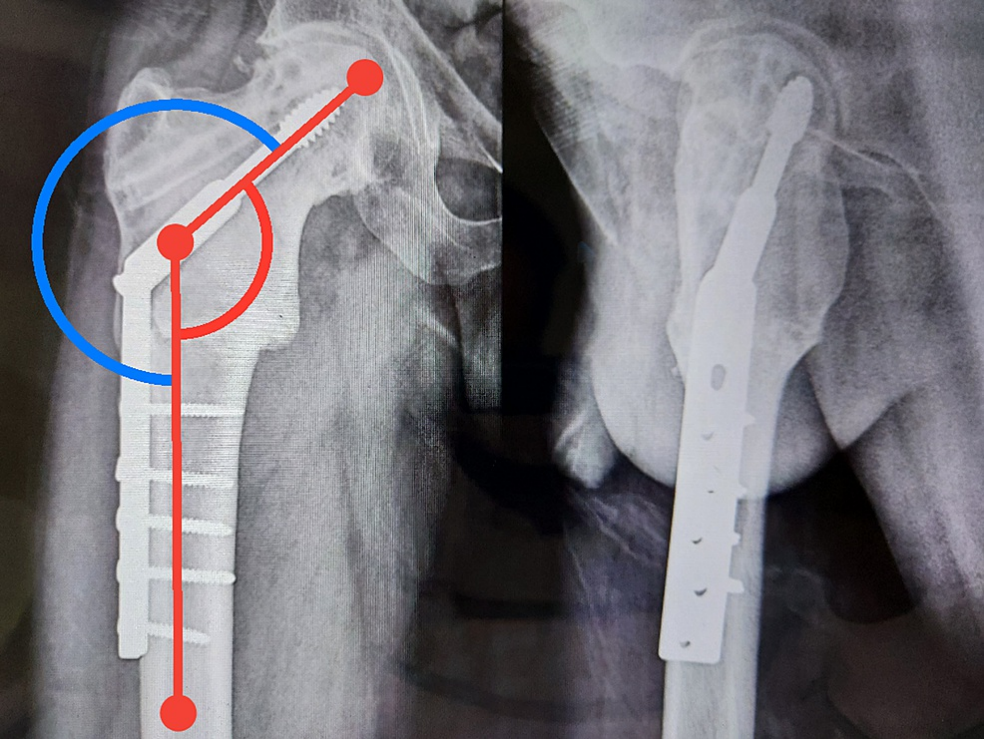
Follow-up x-ray after six months with the restored neck-shaft angle of 130 degrees

The patient was followed up in the outpatient department (OPD), and x-rays were taken. Radiological sign of healing was observed at three months. Partial weight-bearing was allowed at three months and full weight-bearing at six months with no restriction in the activity. After six months, the patient was able to perform all activities without any difficulty, and the shortening of 1.5 cm was compensated with footwear modification. No evidence of recurrence was noted in the follow-up x-ray (Figure 3).

## Discussion

FD being a benign lesion may go undetected and may present as a deformity like a shepherd's crook as observed in our patient, which was affecting the quality of life and function of the joint. The presentation in our patient was in the third decade, and the patient had neglected his symptoms for six months. The patient was walking full weight-bearing in spite of his injury with a limp. Each Shepherd's crook deformity presents in a different way and with a variety of challenges, thus demanding patient-specific approaches. Customized approaches are needed to achieve correct alignment so as to obtain optimal functional outcomes and better quality of life for the patient.

In our case, Shepherd's crook deformity was associated with a pathological neck femur fracture, so it warranted the use of a dynamic implant like a DHS with a vascularized fibula graft. DHS provides controlled collapse of fracture at the proximal femur, which was aptly supported by the fibular strut graft. We prefer the technique described by Li et al. [10] who concluded that “valgus osteotomy in combination with DHS internal fixation is an easy and effective method for the treatment of FD with Shepherd's crook deformity as it can restore the neck-shaft angle and re-establish the mechanical alignment of the femur to improve function.” Also, a desired length of the side plate depending on the extent of the lesion helps to span the lesion completely and prevent a recurrence.

The aim of the osteotomy should be to achieve neck-shaft alignment as well as proximal femur and shaft alignment and address issues such as fracture neck femur as in our case. The union of osteotomy is a risk, but proper mechanical alignment union is usually certain. The site of osteotomy depends on the extent of the lesion in the proximal femur. The single-stage correction of Shepherd's crook deformity helps in early rehabilitation and decreases patient morbidity.

## Conclusions

Valgus osteotomy is an effective method to achieve mechanical alignment, correct neck-shaft angle, and address pathological neck femur fracture. The use of DHSs allows for collapse at the fracture site and spans the barrel plate according to the lesion. Cortical strut graft has also been used to additionally support the neck femur fracture. Single-stage surgery has the advantage of early rehabilitation and better patient outcomes. Good curettage with proper mechanical alignment also reduces the chances of recurrences.
